# Decoding regulatory associations of G-quadruplex with epigenetic and transcriptomic functional components

**DOI:** 10.3389/fgene.2022.957023

**Published:** 2022-08-25

**Authors:** Shuyi Fang, Sheng Liu, Danzhou Yang, Lei Yang, Chang-Deng Hu, Jun Wan

**Affiliations:** ^1^ Department of BioHealth Informatics, Indiana University School of Informatics and Computing, Indiana University—Purdue University Indianapolis, Indianapolis, IN, United States; ^2^ Department of Medical and Molecular Genetics, Indiana University School of Medicine, Indianapolis, IN, United States; ^3^ The Collaborative Core for Cancer Bioinformatics (C^3^B) shared by Indiana University Simon Comprehensive Cancer Center and Purdue University Center for Cancer Research, Indianapolis, IN, United States; ^4^ Department of Medicinal Chemistry and Molecular Pharmacology, Purdue University, West Lafayette, IN, United States; ^5^ Purdue University Center for Cancer Research, Purdue University, West Lafayette, IN, United States; ^6^ Department of Pediatrics, Indiana University School of Medicine, Indianapolis, IN, United States; ^7^ Herman B Wells Center for Pediatric Research, Indiana University School of Medicine, Indianapolis, IN, United States; ^8^ Center for Computational Biology and Bioinformatics, Indiana University School of Medicine, Indianapolis, IN, United States

**Keywords:** G-quadruplex, G4 feet, transcription, gene expression, enhancer, DNA methylation, open chromatin, motif

## Abstract

G-quadruplex (G4) has been previously observed to be associated with gene expression. In this study, we performed integrative analysis on G4 multi-omics data from in-silicon prediction and ChIP-seq in human genome. Potential G4 sites were classified into three distinguished groups, such as one group of high-confidence G4-forming locations (G4-II) and groups only containing either ChIP-seq detected G4s (G4-I) or predicted G4 motif candidates (G4-III). We explored the associations of different-confidence G4 groups with other epigenetic regulatory elements, including CpG islands, chromatin status, enhancers, super-enhancers, G4 locations compared to the genes, and DNA methylation. Our elastic net regression model revealed that G4 structures could correlate with gene expression in two opposite ways depending on their locations to the genes as well as G4-forming DNA strand. Some transcription factors were identified to be over-represented with G4 emergence. The motif analysis discovered distinct consensus sequences enriched in the G4 feet, the flanking regions of two groups of G4s. We found high GC content in the feet of high-confidence G4s (G4-II) when compared to high TA content in solely predicted G4 feet of G4-III. Overall, we uncovered the comprehensive associations of G4 formations or predictions with other epigenetic and transcriptional elements which potentially coordinate gene transcription.

## Introduction

G-quadruplexes, which are also called G4 structures, are non-canonical secondary organizations formed by folding of guanine-rich DNA or RNA sequences (Spiegel et al., 2020). Given the four-stranded structure, G4 formations are diverse in strand direction, length and loop composition (Burge et al., 2006; Zhou et al., 2012). They were identified to be associated with specific biological processes at both transcriptional and epigenetic levels. For example, G4 formation was related to gene expression at specific chromatin locations and interacted with other regulatory mechanisms (Du et al., 2008; Rhodes and Lipps, 2015). G4 structures were mainly enriched at the boundaries of topological associated domains (TADs) with important roles in aging, cell differentiation, and cell fate determination (Hou et al., 2019). A DNA methyltransferase enzyme, DNMT1, was found to have stronger interaction with G4s than with duplex DNA. G4 formation reduced enzyme activity of DNMT1, resulting in decreased methylation of CpG islands (CGIs) on DNA (Mao et al., 2018). Meanwhile, DNA methylation also influenced the topology of G4 structures and binding activities of G4s with other proteins (Tsukakoshi et al., 2018). On the whole genome, G4s were concentrated at multiple specific genome locations, such as telomeres, the first intron of genes and gene regulatory regions including promoters, 5′ untranslated regions and splicing sites (Du et al., 2009; Maizels and Gray, 2013). Many telomere-associated proteins could interact with G4 structures to prevent telomerase-mediated extensions at the telomeric ends and then repress cell growth (Sun et al., 1997; Fouquerel et al., 2016).

Currently, the underlying molecular mechanisms of G4 on gene regulation remain largely elusive. Accumulated evidence suggests G4s might play dual roles in regulating gene expression. Some previous studies showed that genes with G4 formation on the promoters were suppressed, including many proto-oncogenes, such as MYC and KRAS (Siddiqui-Jain et al., 2002; Cogoi and Xodo, 2006), and genes related to DNA damage, cell differentiation, such as UCP1 (Zhao and Uhler, 2018). However, other studies (Hansel-Hertsch et al., 2016; Robinson et al., 2021) found that G4 could be related to gene activation and trigger corresponding pathways. For instance, G4s were found enriched around cancer-promoting genes in cancer cells compared to normal ones, while G4 activities caused DNA double-strand breaks in cancer cells and activated DNA repair pathways (Paeschke et al., 2013). To identify genome-wide G4 formations and explore their functions, many technologies, especially next-generation sequencing (NGS), have been employed to detect or predict the locations and sequences of G4s for the different sequence structures compared to double-stranded DNA or RNA. For instance, G4-seq is a high-resolution sequencing-based method to detect G4 structures in the human genome, which identified more than 700,000 G4 sites (Chambers et al., 2015). Since G4 structures can be specifically bound by serval proteins, including BG4 (Hansel-Hertsch et al., 2016), 1H6 (Mao et al., 2018), D1^14^ and artificial G4 probe (G4P) protein (Zheng et al., 2020), chromatin immunoprecipitation followed by high-throughput DNA sequencing (ChIP-seq) using these specific antibodies was designed to detect genome-wide G4 sites. Of course, different antibodies in discrete cell lines resulted in diverse G4 genomic locations, suggesting binding specificity of the antibody to the G4 structures as well as dynamic G4 formations in different cell lines. However, the majority of G4 sites were found consistently in similar regions which are connected to important biological functions and processes.

**FIGURE 1:**
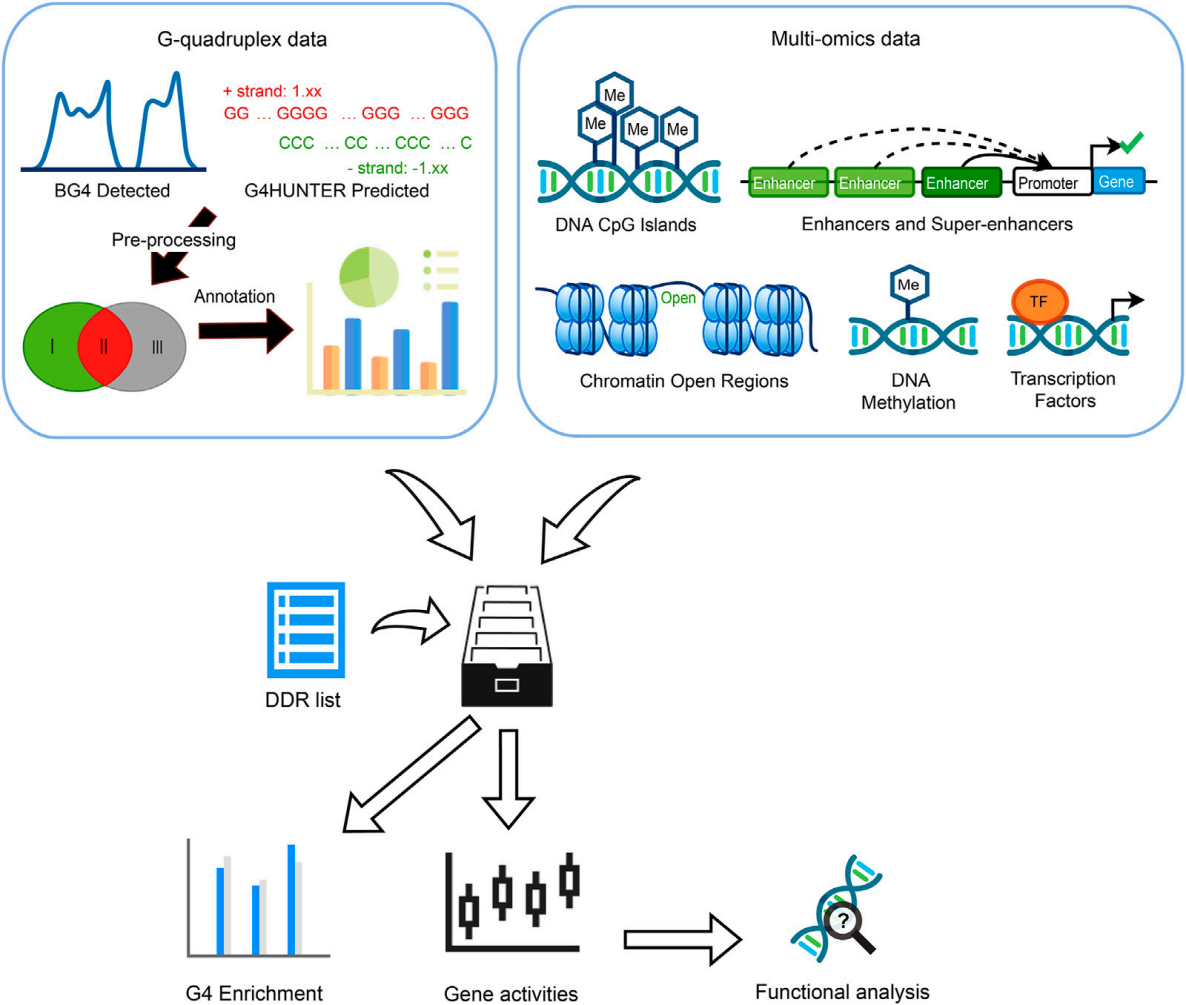
Overview of the whole study. Three G4 groups were defined by evidence collected from G4 ChIP-seq and G4Hunter prediction. Multi-omics data including CpG islands, enhancers, super-enhancers (SEs), chromatin accessible/inaccessible regions, DNA methylation as well as transcription factors (TFs) binding sites were incorporated to discover the G4 enrichment, associations between gene expression, G4 formation, and epigenetic regulators.

In this study, we incorporated G4 prediction method and experimental G4 ChIP-seq results to classify G4 sites into three groups based on the experimental evidence and computational prediction (Figure 1). The G4 enrichment analysis was conducted in categorized genome locations to compare their potential associations with gene regulation. Incorporating multi-omics data, we explored the relationships of gene expressions with G4 groups and other epigenetic or transcriptomic functional components, such as CpG islands (CGIs), enhancer and super-enhancer (SE) regions, open chromatin regions, and DNA methylation. We employed an elastic net regression model to estimate the contributions of G4 structures and other well-known epigenetic regulators to gene expression detected. The examination on overlapping between transcription factor (TF) binding sites and G4 groups may indicate the cooperation between G4 and TFs. To further discover potential sequence consensus to support or prevent G4 formations, motif analysis was performed in the G4 feet, flanking regions of two groups of G4s. Two sets of recognizable motifs were identified with significant enrichment in two groups, respectively, with different GC contents. In general, our results revealed the distinct roles of three G4 groups given different evidence levels, from experiments and/or based on in-silicon prediction, suggesting potential biological functions related to G4 formation. This study shed more insights into the relationship between G4s and multiple epigenetic regulators which are tightly linked with transcriptional activities and gene expression.

## Results

### Groups of G4 structures classified by evidence

**FIGURE 2:**
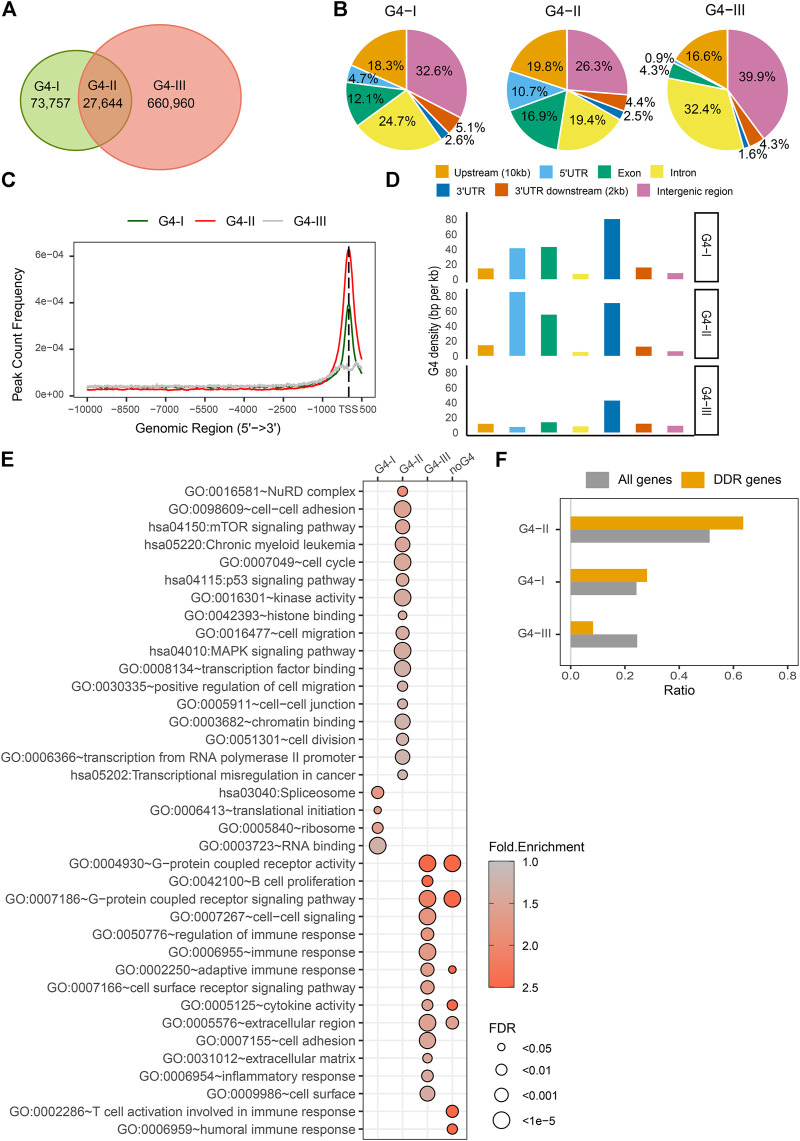
Distinct features and biological functions of three G4 groups. **(A)** Numbers of G4 regions confirmed by G4 ChIP-seq only (G4-I) or confirmed by the ChIP-seq and predicted by G4Hunter (G4-II), or only predicted by the G4Hunter (G4-III). **(B)** Distributions of three G4 groups in genome locations, including upstream up to 10 kb from gene transcription start sites (TSSs), 5′UTR, exons, introns, 3′UTR, 2 kb downstream of 3′UTR, and intergenic region. **(C)** Distances of different G4 groups from TSS. **(D)** G4 density (BPKB) in different genome locations for three groups. **(E)** Significantly enriched GO terms and KEGG pathways in the gene sets with different G4 groups or without any G4 sequences in the upstream. **(F)** Ratios of DDR genes associated with three G4 groups compared to all genes.

G4 structures have highly conserved G-rich consensus sequences, G_≥3_N_1–7_G_≥3_N_1–7_G_≥3_N_1–7_G_≥3_ (Huppert and Balasubramanian, 2005; Todd et al., 2005). G4 formation follows the specific folding rules while being affected by a wide range of binding factors (Li et al., 2013). Many computational algorithms have been developed to predict G4 sites genome-widely (Klimentova et al., 2020; Puig Lombardi and Londoño-Vallejo, 2020; Barshai et al., 2021; Rocher et al., 2021), while G4 structures were able to be experimentally identified and mapped on the genome by chromatin immunoprecipitation sequencing (ChIP-seq) with specific G4 antibody (Hansel-Hertsch et al., 2016; Liu et al., 2016). Here we incorporated datasets of G4 regions predicted by the folding rules and experimentally detected in human cancer cells. First, genome locations of over 707,000 predicted G4 sites were collected based on the G4Hunter (Bedrat et al., 2016). Then we incorporated them with G4 ChIP-seq data by an antibody BG4 in K562 cells (Mao et al., 2018). The reason we selected K562 cell line as an example is that most genome-wide data are available for K562 study in the public open database, e.g., ENCODE, including but not limited to G4 ChIP-seq, RNA-seq, DNase-seq, DNA methylation, hundreds of TFs ChIP-seq, and enhancer/super-enhancer, etc., To better understand the common and unique genomic features between experimentally confirmed G4 formations and predicted G4 locations, we separated G4 sites into three groups (Figure 2A). Group G4-I was recognized by G4 ChIP-seq only, whereas group G4-II was both predicted by G4Hunter and confirmed by G4 ChIP-seq which includes 41,857 predicted G4 sites covered by 27,662 binding peaks detected by BG4 antibody in K562 cell line. Group G4-III had a larger number of predicted G4 by G4Hunter, which were not formed in K562 cell line or just not recognized by BG4 ChIP-seq due to the antibody specificity. The groups of G4s showed differences in multiple aspects. Generally, three groups of G4 sites including potential G4s in G4-III had distinct distributions on the genome (Figure 2B). For example, comparing to other two G4 groups, the G4-II group tended to locate at gene upstream up to 10 kb from TSS (19.8%), 5′UTR (10.7%) and exon (16.9%). The G4-III group spread more widely in intron (32.4%) and the intergenic region (39.9%). As shown in Figure 2C, A higher proportion of G4-II sites positioned at and around TSS than those of other two groups, G4-I and G4-III. Particularly, the group of G4-III distributed more about ± 250 bp away from the TSS than at the exact TSS. To avoid biases resulted from either G4 lengths in different groups, or lengths of genomic features annotated here, e.g., exons, introns, etc., we used G4 density (see Methods and Materials) to compare the G4 distribution for three groups at different genome regions. Besides regions close to TSS (Figure 2C), G4-II had higher densities in 5′UTR (85 BPKB), 3′UTR (70 BPKB), and exon (55 BPKB) (Figure 2D), where much sparser G4 sites from the predicted group of G4-III scattered (7 BPKB in 5′UTR, 43 BPKB in 3′UTR, and 13 BPKB in exon).

Next, genes associated with different groups of G4 sites were categorized into three groups and additional one without any G4, which can be used to dissect the associations between G4 structures and gene activities. We extracted gene expression data (RPKM) for K562 cell line from CCLE database, including 17,908 protein-coding genes. Out of them, 17,420 genes had at least one detected or predicted G4 in the 10 kb upstream regions. 8,923 (51.2%) of them were defined as G4-II genes which had at least one G4-II site, while 4,226 (24.3%) genes had at least one G4-I but no G4-II named as G4-I genes. Other 4,271 (24.5%) genes associated with predicted G4-III only in their upstream regions were called G4-III genes. 488 genes with neither predicted nor detected G4 upstream were distinguished from others. Generally, these four groups of genes were over-represented for different Gene Ontology (GO) (Ashburner et al., 2000; Gene Ontology Consortium, 2021) biological processes or molecular functions or in different KEGG pathways (Figure 2E). For instance, genes with G4-II in the upstream were notably enriched in the pathway of chronic myeloid leukemia because the G4 ChIP-seq data was collected from the study that based on K562 cell line (Mao et al., 2018). G4-II genes were also implicated in mTOR, p53, and MAPK signaling pathways. They were significantly over-represented in NuRD complex and cell-cell junction/adhesion, while being involved in cell cycle, cell division, cell migration, especially positive regulation of cell migration. G4-II genes were active in transcriptional dysregulation in cancer *via* different molecular functions, e.g., histone binding, chromatin binding, transcription factor binding, transcription from RNA polymerase II promoter, and even kinase activity. Genes with G4s identified by ChIP-seq only (G4-I) were observed associated with RNA binding and translation initiation. They tended to locate on ribosome and spliceosome. As a comparison, genes with predicted G4s which were not detected by ChIP-seq (G4-III) or genes without any G4 in upstream were enriched in extracellular regions and cell surface. Those G4-III genes were also implicated in immune response, including regulation of immune response, adaptive immune response, B cell proliferation, inflammatory response, besides G-protein coupled receptor activity, cell-cell signaling, and cell surface receptor signaling pathway. Genes without any groups of G4s as defined seemed specific to T cell activation involved in immune response and humoral immune response while sharing some biological functions and pathways with G4-III genes together.

It has been reported (Robinson et al., 2021) that G4 sites were related to DNA damage response (DDR). We collected DDR genes from GO database according to the keywords in their associated GO terms, including “double-strand break repair” or “DNA damage response.” A total 327 DDR genes were found to have G4 sites from 10 kb upstream to TSS, in either group of G4-I, G4-II, or G4-III. Among them, 208 (63.6%) genes had at least one G4 structure predicted as well as confirmed by the ChIP-seq (G4-II) (Figure 2F). The ratio was notably (*p* = 3.4 × 10^–6^) higher than that of all genes with at least one G4-II (51.2%), suggesting that G4-II sites were apt to be over-represented in the upstream of DDR genes. A similar but lower level of over-representation was observed in DDR genes with G4-I but without G4-II, while very fewer DDR genes had predicted G4-III only (Figure 2F).

### Distinct transcription activities associated with different groups of G4s

**FIGURE 3:**
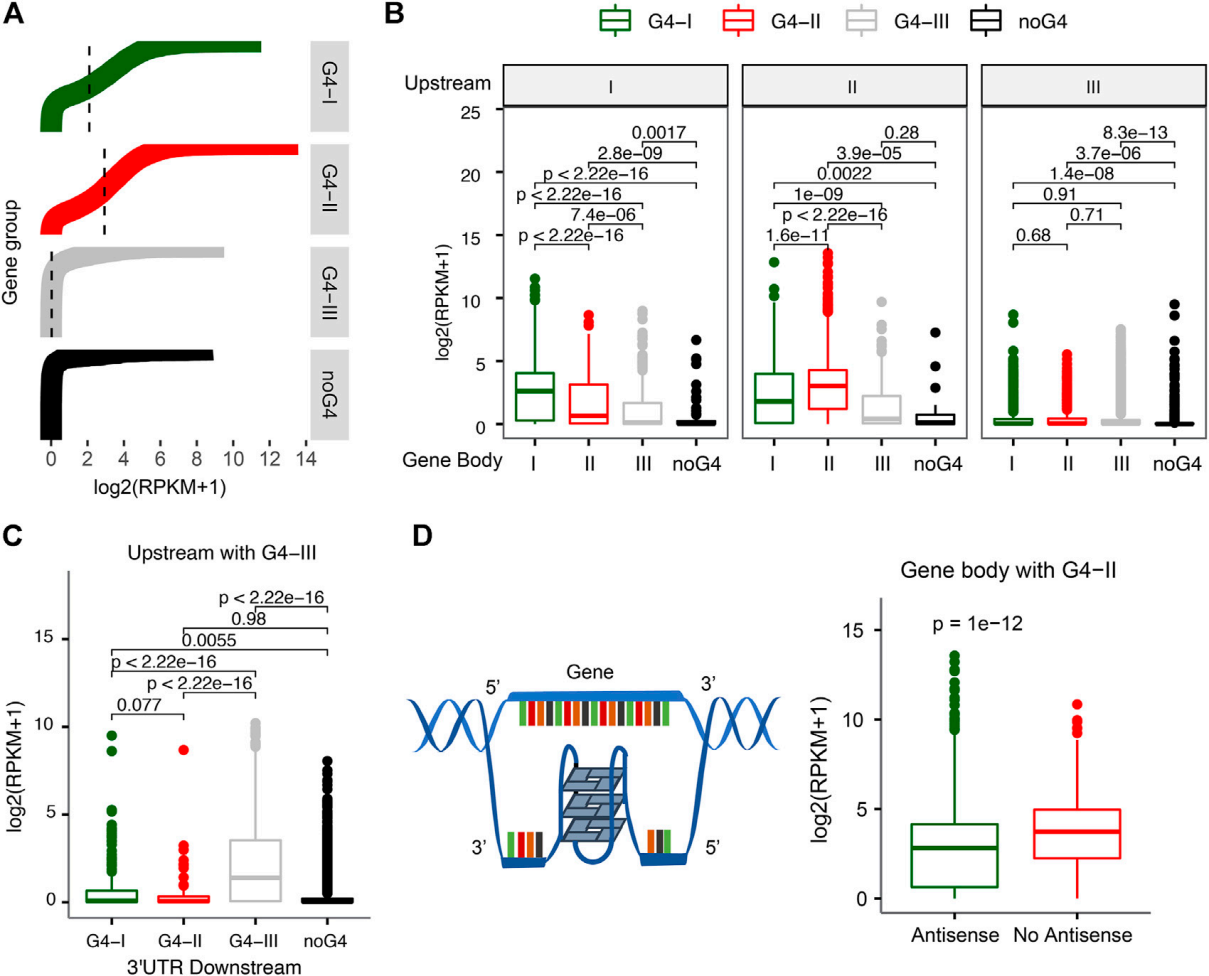
Relationship between gene expression and different G4 groups. **(A)** Distinct expression levels for genes with divergent G4 groups (G4-I, G4-II, and G4-III) or without G4 in the upstream. **(B)** Differences of gene expression levels with G4 combinations between the upstream and gene body. **(C)** G4-III in both upstream and 3′UTR downstream leading to higher gene expression levels. **(D)** Decreasing expression levels for genes with G4-II on the antisense of gene body.

The distributions of G4s in gene upstream and 5′UTR, along with GO functions and pathways related to transcription regulation identified enriched in G4-II genes (Figure 2D), suggest that G4 structures might have strong associations with gene transcription activities. Indeed, genes associated with different G4 groups were found with marked expression profiles (Figure 3A). Genes with G4-II in upstream regions tended to have the higher expression levels, followed by genes with G4-I but without G4-II (Figure 3A). The median expression level for G4-II genes was about 1.5-fold of genes with G4-I but without G4-II (*p* < 2.2 × 10^–16^). About 22% of 4,271 genes with only predicted G4-III were not expressed in K562 cell line. Genes without any G4 at upstream regions had the lowest mean expression level even compared to genes with G4-III only (*p* = 2.4 × 10^–15^). The consistent patterns were observed in another cell line, A549 cells, as well (Supplementary Figures S1A, B), where G4 probe (G4P) was used with very high affinity and specificity for G4 binding to identify G4 sites in living cells (Zheng et al., 2020). These results suggest strong connections between G4 structures and activation of gene expression.

We also examined whether G4s on gene body instead of gene upstream could be associated with different levels of gene expression. It turned out that genes with either G4-II or G4-I on the gene body showed higher expression levels than genes with G4-III or without G4 (Supplementary Figure S2). Then we combined G4 locations (or no G4) for both upstream and gene body or downstream. The higher expression levels were noticed for genes with the same evidence-determined group of G4 on both upstream and gene body (Figure 3B). For instance, genes with G4-I on upstream as well as gene body had higher expression levels than genes with G4-I on upstream but with other groups of G4s, like G4-II or G4-III, or without G4 on gene body (left panel in Figure 3B). Similar results were obtained for genes with G4-II in upstream (middle panel in Figure 3B). However, genes with G4-III in upstream did not show notable changes no matter the genes had either G4-I/II/III groups or no G4 on gene body. It was surprising to see those genes with G4-III in both upstream and 2 kb downstream from 3′UTR tended to express highly (Figure 3C). But no significant expression difference was observed for genes with either G4-I or G4-II in both upstream and 2 kb downstream.

Next, we focused on genes with the highest confidence G4 formation, G4-II, on gene body to investigate the relationship between gene expression and G4 locations on two DNA strands. Genes with G4(s) on gene body were divided into two groups depending on the location of G4(s) on the antisense or the sense strand (Figure 3D). We found that genes with G4-II existing on the antisense had 1.35-fold lower expression levels than those without any G4-II on the antisense (Figure 3D). This is in line with the transcription process because the antisense of the gene serves as a non-coding RNA template for synthesis of a complementary transcript. The high-order G4 structures on the antisense/template might become blocks to potentially reduce or even pause the transcript processing then consequently repress the gene expression.

### Correlations between G4 groups, epigenetic elements, and gene expression

**FIGURE 4:**
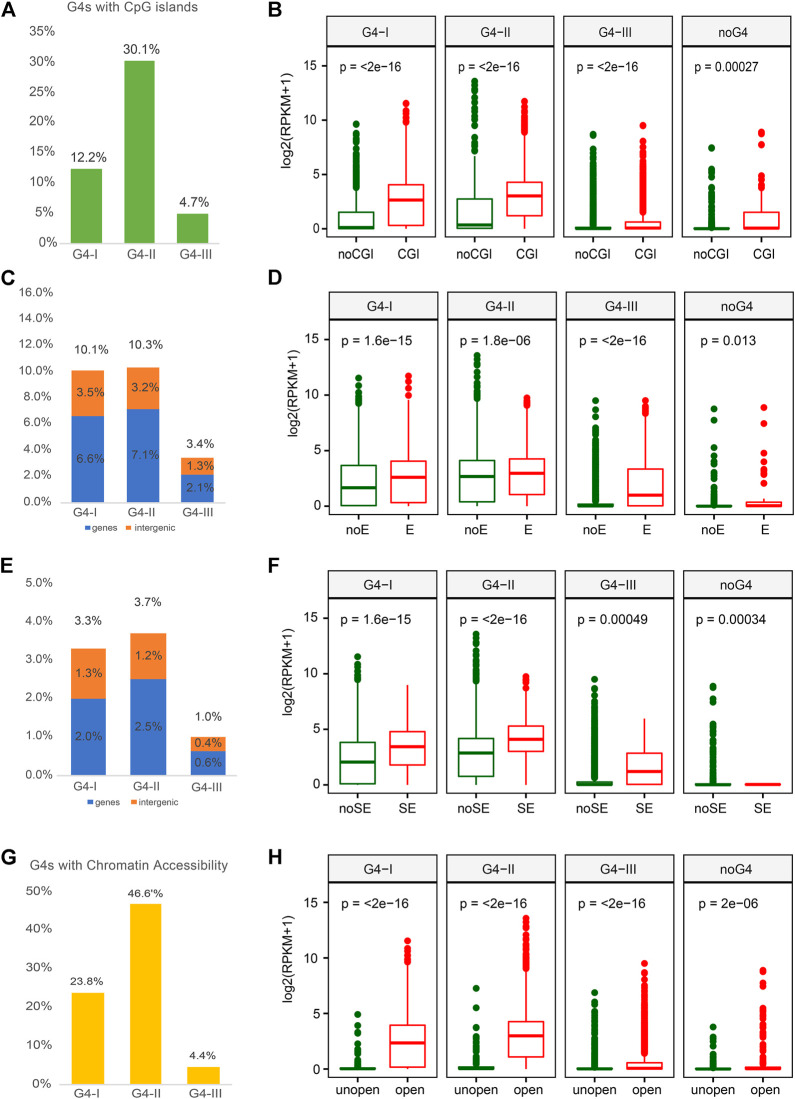
Overlaps between G4 groups and multiple epigenetic regulatory regions with corresponding gene expression. **(A)** Ratios of G4 groups overlapping with CpG island (CGI) and. **(B)** expression profiles for genes with three G4 groups or without any G4 in or out of CGI. **(C)** Ratios of G4 groups overlapping with enhancer and. **(D)** expression profiles for genes with different G4 groups or without any G4 in or out of enhancer. **(E)** Ratios of G4 groups overlapping with SE and. **(F)** expression profiles for corresponding genes comparing to the SE. **(G)** Ratios of G4 groups overlapping with open chromatin regions and. **(H)** expression profiles for genes with three G4 groups or without any G4 in or out of chromatin open regions.

As CpG enriched regions occupy over half of promoter sequences, CGIs are important for transcription regulation (Steinhaus et al., 2020). Previous study also reported that G4 formations were related to CGIs with lower methylation level (Mao et al., 2018). Thus, we next explored the relationship between different groups of G4s and CGI, which might be able to help us understand the roles of G4s in gene expression. Over 30% of G4-II were found to locate in or overlap with CGIs, which was higher than the colocalization ratios of G4-I (12%) and G4-III (5%) (Figure 4A). Elevated expression levels of genes with G4 in CGI were observed compared to those genes with G4 outside of CGI (Figure 4B). Furthermore, some studies reported that G4 structures were strongly associated with enhancer activity (Hegyi, 2015; Hou et al., 2019). In this case, we collected enhancer and super-enhancer (SE) data from Super-Enhancer Archive (SEA) (Chen et al., 2020) to examine whether G4s had a preference for such active regions. Both groups of G4-II and G4-I were prone to colocalize with enhancers in K562 cells, 10.3%, and 10.1%, respectively (Figure 4C). However, G4-I was inclined to locate in more intergenic regions compared to G4-II (Figure 4C). Considering the function of enhancers, it is not surprised to see genes with G4s within enhancers expressed more highly than those with G4s outside of enhancers, especially for G4-III (Figure 4D). A similar trend for G4 locating within or outside of SEs was also perceived (Figure 4E). G4s within SEs were apt to be associated with genes whose expression levels were about two-fold higher for other genes whose G4s either confirmed or predicted only were not located within SEs (Figure 4F).

Another factor that is directly linked to gene expression is TF activity that can be impacted by the chromatin status (Klemm et al., 2019). Normally open chromatin regions with nucleosome depletion allow TFs to bind to specific DNAs then regulate the expression of downstream genes (Schones et al., 2008). To understand the correlation between G4 formation and gene expression, we used DNase-seq data in K562 from the ENCODE to investigate the formation preference of G4 structures in open chromatin regions. The group of G4-II was observed with the highest ratio in open chromatin regions compared to both G4-I and G4-III (Figure 4G). About half (46.6%) of G4-II was detected in the nucleosome-depleted regions, while only one-quarter of G4-I and less than 5% of G4-III were found to be overlapped with open chromatin regions identified by the DNase-seq. These suggest that the G4 structures were likely formed in nucleosome-depleted regions, or might tend to assist chromatin open, to interact with gene regulatory elements and TFs. Particularly, genes with G4s, especially G4-II, in open chromatin regions were prone for higher expression levels (Figure 4H).

The above results indicate collectively that G4s had strong associations with gene expression by working together with multiple epigenetic regulatory elements, whereas different evidence based G4 groups showed distinct effects, such as G4-II genes being connected with higher gene activations. To further explore the relationships among gene regulation and all functional components above, including three G4 groups in the upstream of genes (“up”) CGI, enhancer SE, open chromatin, and G4s locating on the gene antisense, we employed an elastic net regression model (Zou and Hastie, 2005; Friedman et al., 2010) to predict the gene expression into two categories, high and low (see Methods for more details). The final model was presented as

$$y=-4.94+0.80\delta_{G4-I:up}+1.60\delta_{G4-II:up}-1.21\delta_{G4-III:up}+0.56\delta_{Enhancer}+1.33\delta_{SE}+1.73\delta_{CGI}+3.00\delta_{OpenChrom}-0.15\delta_{G4:Antisense,}$$

where *y* is the log-odds of an event that gene is highly expressed and $\delta_{i}$ represents the existence of individual feature *i*,

$$\delta_{i}=\{\begin{array}{cc} 1, & feature\;i\;exists\\ 0, & none \end{array}$$

**FIGURE 5:**
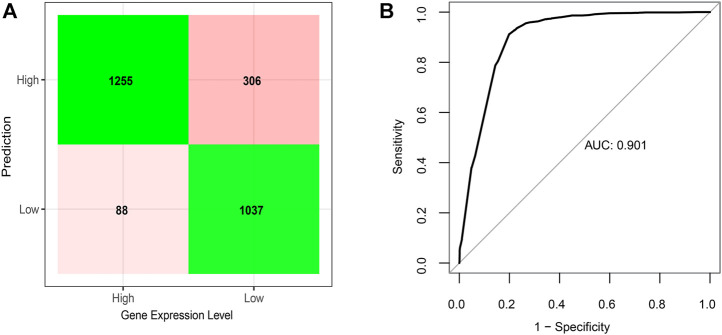
Elastic net regression model on gene expression predicted given the status of G4 formation and location, CGI, enhancer, super-enhancer, and chromatin status. **(A)** Confusion matrix of prediction results based on the final regression model on testing dataset. **(B)** Corresponding ROC curve with AUC score of the regression model.

Consistent with our observations according to individual comparisons aforementioned, the model showed positive coefficients for features, G4-I or G4-II formed in the upstream of genes, the presence of enhancer or SE, existence of CGI, as well as open chromatin status, indicating their positive correlations with downstream gene expression. In contrast, two other features, predicted-only G4-III in the gene upstream and G4 formed on the gene antisense, emerged with negative coefficients in the model, suggesting that they were related to lower gene expression. The model could successfully predict expression levels for 85% genes (Figure 5A), which can reach an area under curve (AUC) score of 0.90 (Figure 5B).

### Relationship between G4 structure and DNA methylation

DNA methylation may influence gene expression by interrupting TF binding activities (Lister et al., 2009; Medvedeva et al., 2014; Ambrosi et al., 2017; Héberlé and Bardet, 2019). The formation of G4s was induced more often in G-rich genomic regions, suggesting a relation between G4 structure and DNA methylation. It has been reported that G4 structure inhibited local methylation by protecting CpG islands from methylation (Mao et al., 2018).

**FIGURE 6:**
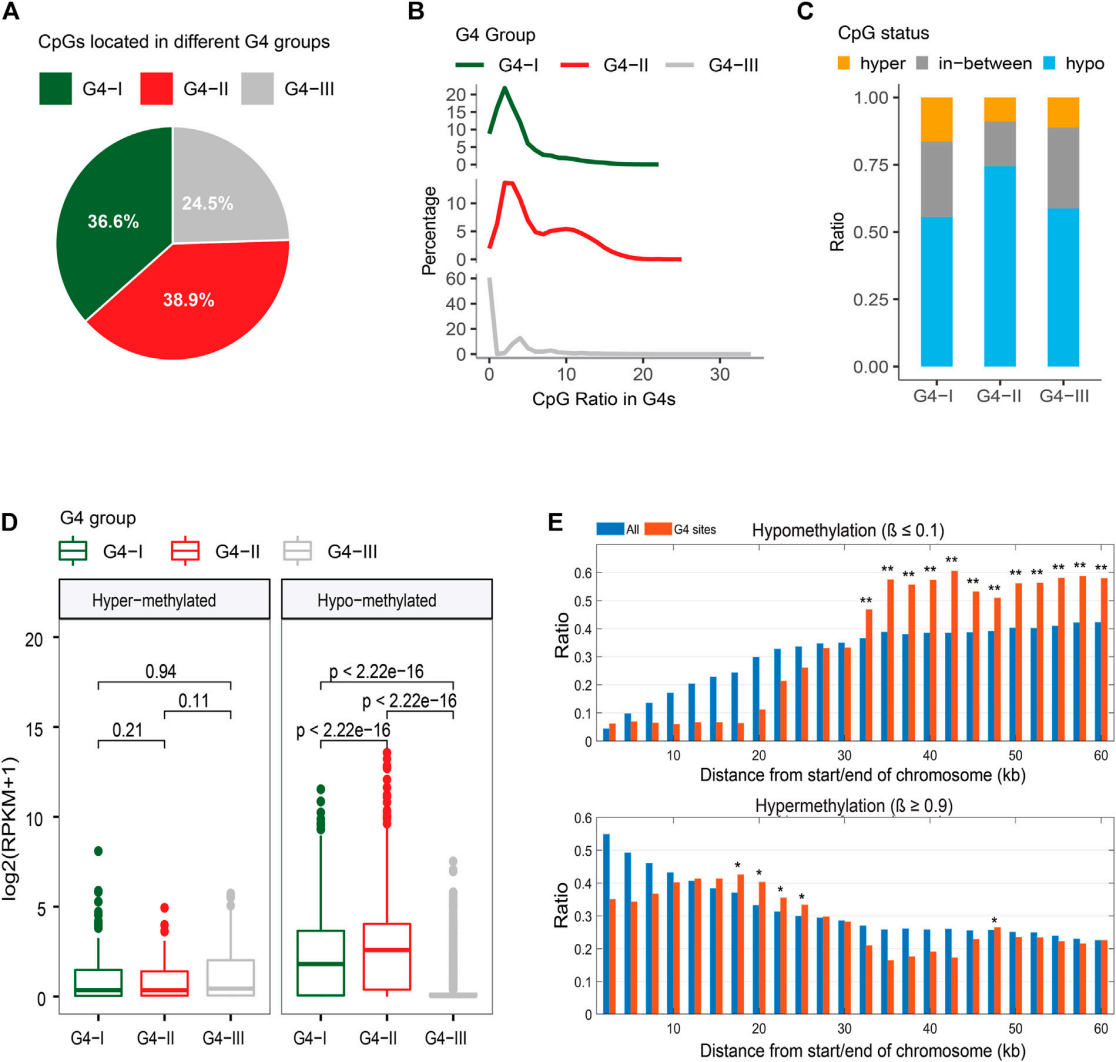
Relationship between G4 groups and DNA methylation. **(A)** Distributions of CpG sites locating in three G4 groups. **(B)** CpG density distributions in G4 groups. **(C)** Distinct CpG methylation levels in three G4 groups. **(D)** Expression levels of genes with upstream G4s containing only hypermethylated or only hypomethylated CpG sites. **(E)** DNA methylation patterns within or close to telomeres for all CpG sites and CpG sites covered by G4 sites only.

We extracted DNA methylation data in K562 from the ENCODE database (ENCODE Project Consortium, 2012; Davis et al., 2018) then integrated them with the information of G4 groups in our study. A total of 1,412,581 CpG sites were covered by ChIP-seq-confirmed or predicted G4s with at least 10 sequence-read-counts achieved by the bisulfite-seq. About 36.6%, 38.9%, and 24.5% of CpG sites of our selection located in groups G4-I, G4-II, and G4-III, respectively (Figure 6A). Considering the G4 numbers and lengths in three groups, G4-II indicated the highest coverage of CpG compared to G4-I and G4-III (Figure 6B). The genome-wide analysis showed that 63.7% of CpG sites were hypomethylated (β ≤ 0.1) while 12.3% of all were hypermethylated (β ≥ 0.9) in K562. It is interesting to see (Figure 6C) that majority (74.4%) of CpG sites in G4-II were hypomethylated, much higher than the ratios of G4-I (55.6%) and G4-III (58.7%). However, 16.4% of G4-I-covering CpG sites were hypermethylated, higher than hypermethylation levels for both G4-II and G4-III. As expected, genes including G4-I or G4-II with hypomethylated CpG sites were expressed at higher levels than those genes with G4s covering hypermethylated CpG sites (Figure 6D). Particularly, genes with hypomethylated CpGs in G4-II were activated more highly than those with G4-I. However, genes with hypermethylated CpGs in predicted-only G4-III had 3.17-fold higher expression levels (*p* = 8.3 × 10^–10^) than genes carrying hypomethylated CpGs covered by G4-III.

Telomeres are genome sequences at the end of chromosomes that potentially protect the chromosome ends from sequence degradation and fusion with other ones. Telomeres were reported as G-rich in nucleotides (Wright et al., 1997), subsequently, DNA sequences on telomeres have a potential to form G4 structures. Within telomeres and genome regions close to the telomeres (equal to or less than 60 kb from the end of chromosomes selected in the study), the DNA methylation patterns for CpG sites within potential G4 structures were obtained very differently from those for all CpG sites (Figure 6E). Even though fewer CpGs exist within 10 kb from the chromosome ends, both ratios of hypo-and hyper-methylated CpGs were much lower within those G4s. However, CpG hypermethylation ratios increased on the G4s around 20 kb from the chromosome ends, whereas significant upward flight of CpG hypomethylation ratios was observed on the G4s from 30 kb to 60 kb.

### Potential cooperation between G4 structures and TFs

**FIGURE 7:**
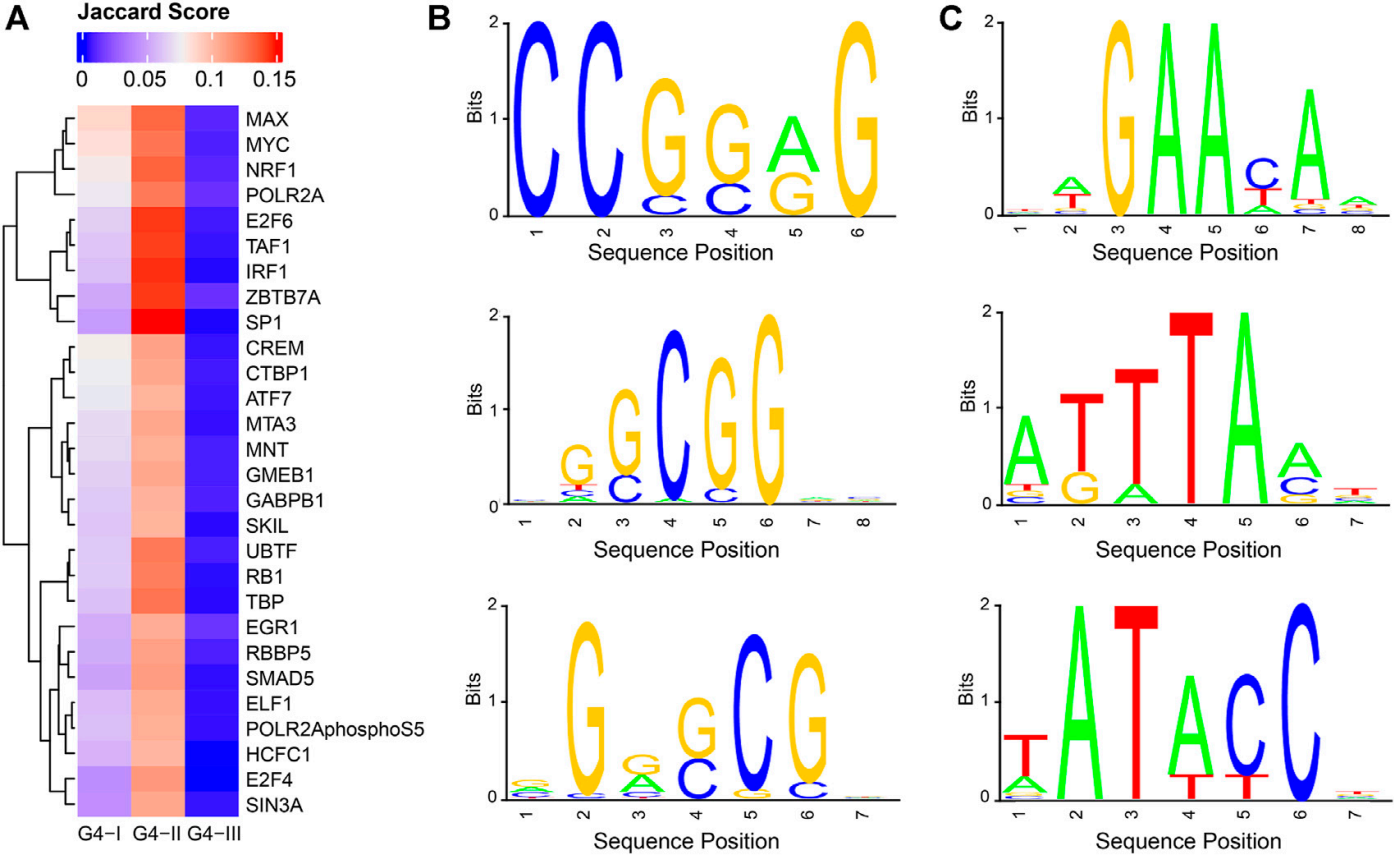
Potential cooperation between G4 groups and TFs as well as novel consensus sequences identified in G4 feet. **(A)** Jaccard scores to evaluate the overlapping ratios between binding sites of TFs and G4 groups. 28 TF s were selected with Jaccard scores larger than 0.1 for at least one of three G4 groups. **(B)** Three selected sequence logos enriched in G4-II feet. **(C)** Three selected sequence logos identified for G4-III feet.

From perspectives of gene expression and biological functions associated with genes having G4 structures, we observed clear connections between G4 and gene transcription. To explore TFs which might potentially co-work with G4 structures, we collected ChIP-seq data for 205 TFs in K562 (Moore et al., 2020) (Supplementary Table S1) to identify their binding peaks, separately, followed by Jaccard score calculations to evaluate the overlaps between the binding sites of each TF and different groups of G4s (Supplementary Table S2, Figure 7A). It showed that Jaccard scores of most TFs in G4-I and G4-II were evidently higher than those with G4-III. This might be due to much larger numbers of G4-III sites than those of G4-I and G4-II. It was also consistent with our observation that predicted G4-III tended to locate in introns and intergenic regions indicting the lower overlapping between G4-III and TFs. 28 TFs exhibited Jaccard scores higher than 0.1 with G4B (Figure 2D), much larger than the maximum scores of all TFs with G4-I (0.087) and G4-III (0.013). Among them, TAF1 and POLR2A are two genes playing pivotal roles in RNA polymerase activities, in addition to another upstream binding TF, named UBTF, which is associated with ribosomal RNA transcription and chromatin remodeling. Some TFs with cancer-related biological functions also showed high Jaccard scores with G4-II, such as E2F6, IRF1, and MYC. Combined with functional enrichment analysis on G4 genes, these results suggest again the strong associations between transcription activities and G4 structures, particularly G4-II predicted and confirmed by ChIP-seq.

Assuming G4-II had the highest confidence on G4 formation given the evidence from both prediction and experiment, we asked another question: which factors or sequence motifs can potentially facilitate G4 formation? The motif analysis was then conducted on G4 feet, which were defined as flanking regions within 50 bp extension on both sides of G4Hunter predicted DNA sequences. Here we took the highest-confidence G4 formation, G4-II, and predicted-only G4 sequences, G4-III, for the comparison. In terms of the frequencies of 6-mer consensus sequences in G4-II feet and G4-III feet, we found 172 and 278 6-mers significantly (Bonferroni-corrected *p* < 0.05) enriched in G4-II and G4-III feet, respectively (see Methods for more details). For example, GCGCGC, CGGCGC, and CCGCGG occupied about 10% of G4-II feet individually, but less than 1% of G4-III feet, whereas other 6-mers like TATATC and ATAATT appeared in around 0.1% of G4-II feet compared to about 2% of G4-III feet. The motifs identified from G4-II feet were GC enriched (Figure 7B), suggesting the potential roles of these DNA sequences outside the motif regions to assist the G4 formation. In contrast, the feet of G4-III which were not identified by ChIP-seq were significantly over-represented by TA enriched motifs (Figure 7C).

## Discussion

The exact roles and functions of G4 structures with gene expression regulation and corresponding molecular mechanisms with or without other factors still remain largely elusive. In this study, we combined G4 prediction and experimental data in K562 and A549 to define three groups of G4 DNA sequences given such evidence. G4-II group was predicted and identified by G4 ChIP-seq data, whereas the group of G4-I was detected by the ChIP-seq only. G4-I could be either false positive of G4 formation due to the off-target or non-specificity of the antibody, or they were not predicted as G4 sites because of the limitations of the prediction model. However, G4-I was observed to be more similar to G4-II from many perspectives as shown in the results. The other G4 sequences only predicted by computational methods were categorized as G4-III, that were not formed at all in the cell lines we investigated here, or just not targeted by the specific antibody used for ChIP-seq. Previous studies reported that G4 structures were enriched in functional genetic regions such as telomeres and gene regulatory regions (Du et al., 2009; Maizels and Gray, 2013). We found that the group of G4-II, predicted and confirmed experimentally in K562 and A549, preferred to be formed close to TSS, or on exons, or in the regions of CpG islands, enhancers, super-enhancers, and open chromatin regions. The genes with different evidence-based groups of G4s had distinct biological functions and pathways. Genes with G4-II in the upstream were significantly over-represented in chronic myeloid leukemia in K562, in addition to mTOR, p53, and MAPK signaling pathways, NuRD complex, cell-cell junction. These genes orchestrated lots of biological processes, such as cell-cell adhesion, cell cycle, cell division, positive regulation of cell migration, transcriptional dysregulation in cancer, with multiple molecular functions, e.g., histone binding, chromatin binding, transcription factor binding, and kinase activity. Genes carrying G4-I but not G4-II were found enriched in ribosome and spliceosome. They were involved in RNA binding and translation initiation. The predicted-only G4 genes and genes without any G4 shared some biological functions and pathways. They were over-represented in extracellular regions and cell surface, while being involved in immune response, cell-cell signaling, and cell surface receptor signaling pathway.

Lots of previous studies reported that G4s were related to gene repression (Siddiqui-Jain et al., 2002; Cogoi and Xodo, 2006; Marchetti et al., 2018) with different effects and mechanisms. Our biological functional enrichment analysis revealed that genes with G4-II in the upstream participated in transcription regulation, both negatively and positively, which can also be supported by more and more recent studies showing that G4 formation was linked to transcription activation. For instance, the interactions between G4 motif and some proteins were favorable for TF recruitment then further led to downstream gene activation (Tian et al., 2018). Given the gene expression profile in K562, we observed that genes with G4-II both in upstream and on gene body exhibited the highest gene expression levels, followed by genes with G4-I in both regions. The same patterns were observed from another ChIP-seq data using a different G4 antibody (G4P) in a different cell line A549. These indicate a strong association between G4 formation and gene activation, or at least G4-II enrichment preference for highly expressed genes in the functional regions, regardless of the cell lines and antibody used. G4 structures locating on the antisense of gene body were apt to slow down or stop the mRNA transcription, consequently, repress gene expression. Incorporated with other epigenetic regulatory regions, such as CpG islands, enhancers, super-enhancers, and open chromatin regions, genes with G4 locating these regulatory regions showed positive relations with higher expression levels of corresponding genes. Our elastic net regression model indicates the larger positive coefficient for G4-II in gene upstream, following those for open chromatin and CpG island, suggesting stronger correlations between G4 structures and gene activation which might be realized through different epigenetic regulators. With these findings, the detailed mechanisms underlying are worth further collaborations on relative biological experiments.

Moreover, G4s can inhibit local methylation by protecting CpG islands from methylation (Mao et al., 2018). A higher CpG ratio was found in G4-II compared to other two groups of G4s, while G4-II-covering CpG tended to be hypomethylated, which is tightly linked to the higher expression levels of genes with G4-II. We also observed atypical patterns of CpG methylation in G4 sites close to the telomere (within 60 kb), even though the implicit mechanism needs to be explored by more in-depth studies.

Given the observations that G4s, particularly G4-II, were related to higher gene expression, we examined the potential cooperation between G4 structures and multiple TFs or co-factors by calculating the overlaps between G4 sites and binding sites of over 200 TFs identified by ChIP-seq in K562. The notably high overlapping ratios were discerned for TFs playing critical roles in transcription pre-initiation or RNA polymerase activities, e.g., UBTF, TAF1, and POLR2A, in addition to others, like E2F6, IRF1, and MYC, which are involved in cancer-related processes. Further analysis on flanking regions of G4 DNA sequences exhibited GC-content enrichment for G4-II feet compared to TA enriched in predicted-only G4-III feet, suggesting that potential factors recruited by these GC-enriched consensus sequences might be helpful to unwrap the double-strand DNA sequences then support the G4 formation in K562, while those TA-enriched motifs might be able to keep the stability of the DNA double helix to prevent G4 formation. Another scenario is that the GC-enriched DNA sequences around the G4-II might provide more elastic spaces to form G4 structures with possibly extended sequences. Of course, this study was limited by the definition of boundaries of G4 structures identified (see Methods for more details). If the results can be validated by other experiments, such information will be supportive in the detection of G4 structures and help people understand the mechanism of the G4 formation.

In general, the comprehensive analysis on these multi-omics data tried to decode the associations among G4s, multiple epigenetic regulatory factors, and gene expression. Our results may help people understand the important roles of G4 structures, raise the potential of G4 formation as targets in cancer treatment or therapies through diverse regulation activities, although the detailed mechanisms need to be further investigated.

## Materials and methods

### G-quadruplex data collection and annotation

The experimental detected G4 structures were extracted from ChIP-seq data using the BG4 antibody. Data of K562 cell line (GSE107690) was collected from NCBI Gene Expression Omnibus (GEO) (Edgar et al., 2002; Barrett et al., 2012) and pre-processed by a general ChIP-seq analysis pipeline for further investigation. The raw data was firstly trimmed using Cutadapt (Martin, 2011), then aligned with hg19 genome using BWA (Li, 2013), removed from duplicates using Picard (Picard toolkit, 2019), and finally called peaks using MACS2 (Zhang et al., 2008). Furthermore, the ChIP data of G4 probe (G4P) protein in A549 cell line was collected from GEO database with processed peak files (GSE133379).

The predicted G4 regions were generated using R script from G4Hunter (Bedrat et al., 2016) and annotated with hg19 human genome. Detected and predicted G4 regions were separated into three groups for each cell line separately. G4-I group was defined as the G4 regions that were only detected by ChIP-seq but not predicted by G4Hunter. G4-II contains the regions that were both detected by ChIP-seq and predicted by G4Hunter. G4-III was defined as the regions that were only predicted by G4Hunter. All three groups of G4 regions were aligned to UCSC hg19 genome with 10 kb upstream of genes, exons, introns, 3′UTRs, 5′UTRs, 2 kb downstream of 3′UTRs, intergenic regions as well as whole gene body regions (Pei et al., 2022). The distribution pattern from upstream 10 kb to downstream 500 bp region around the TSSs of protein-coding genes was visualized by ChIPSeeker R package (Yu et al., 2015).

### G4 density

Considering the diverse distributions of G4 site lengths in three groups, G4-I/II/III, and different lengths of specific genomic features, we calculated G4 density in the way

$$D_{k}=\frac{\sum_{i\in k}W_{i}}{L_{k}}\times 1,000$$

where *D*_*k*_ is the G4 density (bp per kb, BPKB) in the genome location *k*, such as 10 kb upstream, 5′UTR, exon, intron, 3′UTR, 2 kb downstream of 3′UTR, or intergenic region, which has total length of *L*_*k*_ on the genome, and *W*_*i*_ is the width of *i*th G4 being in the region *k*. Such calculation may avoid the bias from either G4 lengths or lengths of specific genome locations, e.g., exons and introns, of our interests.

### Multi-omics data collection and pre-processing

The gene expression data in K562 and A549 cell lines were collected from Cancer Cell Line Encyclopedia (CCLE) (Ghandi et al., 2019). The list of genes related to DNA damage response functions was generated given their associated Gene Ontology (GO) terms (Ashburner et al., 2000; Gene Ontology Consortium, 2021) with keywords “double-strand break repair” or “DNA damage response.” Both enhancer and super-enhancer (SE) region data in K562 cell line were downloaded from Super-Enhancer Archive (SEA) database (Chen et al., 2020). The uniform 205 transcription factors binding sites (TFBSs), DNase-seq data of chromatin open regions (ENCSR577TXK), and DNA methylation data in K562 cell line (ENCSR765JPC) were all collected from Encyclopedia of DNA Elements (ENCODE) (ENCODE Project Consortium, 2012; Davis et al., 2018).

Enhancer, SE, DNA methylation and TFBS data were all converted from hg38 genome to hg19 version using UCSC LiftOver tool (Kent et al., 2002) to keep analysis consistent, while the DNase-seq data was originally aligned with hg19 genome.

### DNA methylation analysis

DNA methylation data in K562 were downloaded from the ENCODE database (ENCODE Project Consortium, 2012; Davis et al., 2018). The β value, defined as the proportion of methylated CpG/CHG/CHH at individual genome locations detected by the bisulfite-seq, was used to represent the methylation level of corresponding CpG/CHG/CHH site. Because of fewer CHG and CHH sites with strong methylation signals from the whole-genome methylation data, we focused on CpG methylation in this study. Only CpG sites with at least 10 sequence-read-counts achieved from the bisulfite-seq were used to study the relationship between DNA methylation and G4 structure.

### Expression analysis of genes related to G4s

To investigate the expression level of genes related to G4 structures, genes with different groups of G4s in 10 kb upstream regions were marked as different groups. Group of G4-II genes was defined as genes with G4-II, whereas G4-I genes were those with G4-I but no G4-II in the upstream. G4-III genes were genes with only G4-III and the genes without any G4s were named noG4. The genes with groups G4s at downstream of 3′UTR or on the gene body were defined by similar rules.

Taking advantage of the strand information, genes with at least one G4-II at antisense of the gene body were defined as genes with G4-II at antisense. Otherwise, genes were classified as ones without antisense G4-II.

Gene expression levels were compared by boxplots with split panels using ggplot2 R package (Wickham, 2016).

### Elastic net regression analysis

To study the association between G4 formation, selected epigenetic regulatory elements, and gene expression, we applied elastic net regression model with the function of glmnet from R^38^. Considering the categorical features in the model, either existence or nonexistence of three G4 groups, CGI, enhancer, SE, open chromatin, and G4s locating on the gene antisense, we selected the top 25% genes with the highest expression levels and marked their gene expression as the value of 1 (“high”). As an opposite, the bottom 25% genes with the lowest expression level were marked as “low” with the value of 0. These genes were randomly separated into training and test data sets with a scale of 7:3 while the ratio of highly to lowly expressed genes was kept as 1:1. 10-fold cross-validation was used to train the elastic net model. The AUC score of the final model was calculated based on test set with roc function from pROC R package (Robin et al., 2011).

### G4 feet motif analysis

We defined ± 50-bp flanking regions around the G4 sites as G4 feet. The boundaries of G4 sites were defined by G4Hunter prediction. Then we compared the occurrence frequencies of all 6-mer sequences in G4-II feet and G4-III feet, respectively. By comparing the frequencies of 6-mers in the feet of G4-II and G4-III, we evaluated their fold enrichment (F.E.) in either G4-II or G4-III with statistical significance (*p*-value) based on binomial distribution (Wan et al., 2013). All *p*-values were adjusted by Bonferroni multiple-test correction. 172 and 278 6-mers significantly enriched were identified in G4-II and G4-III, separately, with Bonferroni-adj *p* < 0.05, F.E. > 4, and frequency >0.02. These 6-mers were clustered to form motif logos for either G4-II or G4-III feet in the same way as we published (Wan et al., 2013).

### Statistical analysis

Intersection ratios between G4 regions and genomic elements were calculated using the percentage of intersected G4 regions from relative groups. Then statistical significances were evaluated by using hypergeometric model.

Gene expression differences were compared by two-paired T-test.

As for TF binding preference for three G4 groups, the Jaccard scores were calculated to represent overlapping ratios between TFs and different groups of G4 by the bedtools (Quinlan and Hall, 2010).

## Data Availability

The original contributions presented in the study are included in the article/Supplementary Material, further inquiries can be directed to the corresponding author.
