# Supplementary figures and images for: Decoding regulatory associations of G-quadruplex with epigenetic and transcriptomic functional components
Source: Front Genet. 2022 Aug 25;13:957023. doi: 10.3389/fgene.2022.957023 (PMC9452811; doi:10.3389/fgene.2022.957023)

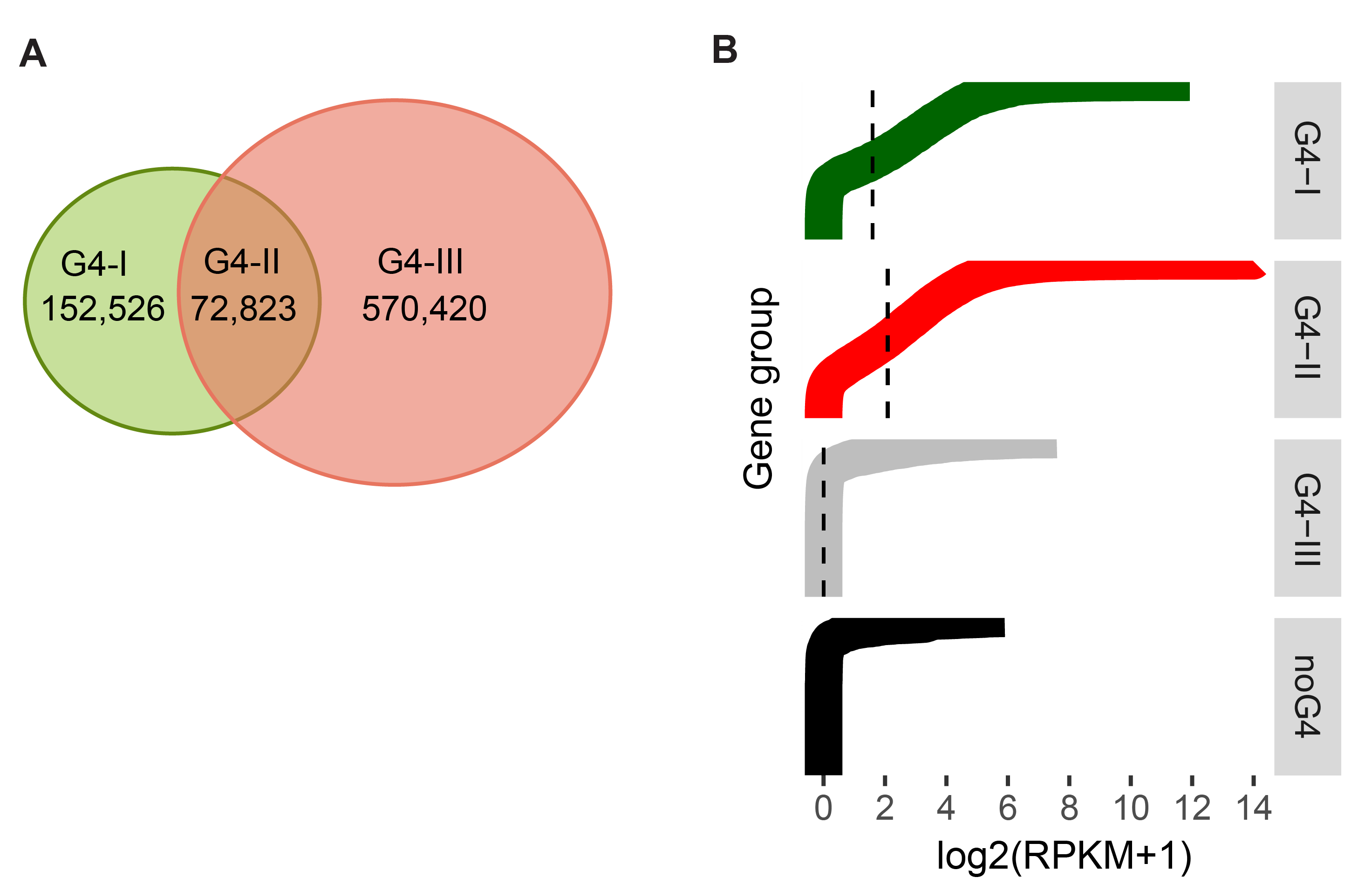

Supplement: Supplementary file 1 [file Image1.tiff]
