# Supplementary material for: Decoding regulatory associations of G-quadruplex with epigenetic and transcriptomic functional components
Source: Front Genet. 2022 Aug 25;13:957023. doi: 10.3389/fgene.2022.957023 (PMC9452811; doi:10.3389/fgene.2022.957023)
Supplement: Supplementary file 2 [file Table1.docx]

**Supplementary Table 1 Resources of ChIP-seq data for 205 TFs in K562 cell line.**

| Dataset ID | file ID | TF |
| --- | --- | --- |
| ENCSR241LIH | ENCFF954QSX | AFF1 |
| ENCSR000EWI | ENCFF910AUJ | SETDB1 |
| ENCSR512NLO | ENCFF119EPK | MNT |
| ENCSR557RVF | ENCFF376CAG | ZHX1 |
| ENCSR000BNN | ENCFF144DMD | THAP1 |
| ENCSR711VWL | ENCFF493LXC | HDAC1 |
| ENCSR000FAW | ENCFF857WHF | POLR2A |
| ENCSR109YGM | ENCFF562ERQ | CREB3L1 |
| ENCSR000BMH | ENCFF938BND | YY1 |
| ENCSR000AQI | ENCFF930OMM | RBBP5 |
| ENCSR000BRK | ENCFF779ZST | TEAD4 |
| ENCSR175EOM | ENCFF045KTH | EHMT2 |
| ENCSR571BUF | ENCFF104IIJ | ARHGAP35 |
| ENCSR893WSB | ENCFF286DHJ | HDAC2 |
| ENCSR491EBY | ENCFF377AEV | ARID2 |
| ENCSR000EGR | ENCFF124MXH | NFYA |
| ENCSR782WRO | ENCFF753LFL | BMI1 |
| ENCSR000FAV | ENCFF181PHR | STAT1 |
| ENCSR000EFU | ENCFF408WSW | ELK1 |
| ENCSR426URK | ENCFF646AZP | AFF1 |
| ENCSR000AQF | ENCFF016MVN | HDAC1 |
| ENCSR000EGE | ENCFF433PKW | EP300 |
| ENCSR657JLK | ENCFF892JRL | SIN3B |
| ENCSR815ZDS | ENCFF869JTA | SREBF1 |
| ENCSR000EGN | ENCFF826HDC | JUND |
| ENCSR201NQZ | ENCFF805RSY | CTBP1 |
| ENCSR530XQI | ENCFF445RNG | L3MBTL2 |
| ENCSR336DXE | ENCFF100VPO | SKIL |
| ENCSR000EWG | ENCFF440KMN | GATA2 |
| ENCSR000EFO | ENCFF317FDT | CUX1 |
| ENCSR986CDX | ENCFF296WNS | NEUROD1 |
| ENCSR099NCH | ENCFF394AJV | ZNF24 |
| ENCSR000EZT | ENCFF090VAI | JUN |
| ENCSR000BNK | ENCFF653WEF | CTCFL |
| ENCSR953DVM | ENCFF008DYZ | E2F8 |
| ENCSR000EGZ | ENCFF457COE | MXI1 |
| ENCSR219BXP | ENCFF181NNL | DPF2 |
| ENCSR091JXL | ENCFF558HHY | HES1 |
| ENCSR000EGM | ENCFF041ODC | CTCF |
| ENCSR343ELW | ENCFF299RMS | LEF1 |
| ENCSR000FCC | ENCFF145JGI | NFE2 |
| ENCSR574XEO | ENCFF909TDT | NUFIP1 |
| ENCSR000AQG | ENCFF660FBQ | HDAC2 |
| ENCSR376WCJ | ENCFF403GZC | IRF2 |
| ENCSR000FAY | ENCFF432VIV | POLR2A |
| ENCSR979QYJ | ENCFF596TWD | MNT |
| ENCSR000EGS | ENCFF838NIW | MYC |
| ENCSR000BNM | ENCFF270QLC | TAF7 |
| ENCSR237VLT | ENCFF571NDF | ZBTB40 |
| ENCSR000BMW | ENCFF809CZV | REST |
| ENCSR000EHL | ENCFF375FRM | POLR2A |
| ENCSR000EHG | ENCFF602YIK | USF2 |
| ENCSR000EFV | ENCFF481AOS | MAX |
| ENCSR000BNV | ENCFF190IHG | MEF2A |
| ENCSR895HSJ | ENCFF438DWU | SMARCA5 |
| ENCSR350XWY | ENCFF825LBQ | C11orf30 |
| ENCSR000EGJ | ENCFF196VRG | MYC |
| ENCSR871TKJ | ENCFF302XKF | THRAP3 |
| ENCSR000BRW | ENCFF528EDV | TRIM28 |
| ENCSR949NVY | ENCFF208AKZ | ZNF639 |
| ENCSR786OQY | ENCFF498TJW | ZBTB5 |
| ENCSR000EWJ | ENCFF680USG | E2F6 |
| ENCSR011NOZ | ENCFF467REJ | ZNF407 |
| ENCSR085DDI | ENCFF408CMK | NFXL1 |
| ENCSR000EGC | ENCFF170JJV | RCOR1 |
| ENCSR643VTW | ENCFF300RMI | SMARCA4 |
| ENCSR808AKZ | ENCFF538UZE | BCOR |
| ENCSR189TRZ | ENCFF883AYB | TCF12 |
| ENCSR000EZX | ENCFF385TGU | JUN |
| ENCSR387UWP | ENCFF410AIK | HDAC1 |
| ENCSR051OUX | ENCFF129NQC | NFATC3 |
| ENCSR000BMG | ENCFF190SJX | HDAC2 |
| ENCSR613NUC | ENCFF446YER | ARNT |
| ENCSR298JCG | ENCFF757UDB | NCOR1 |
| ENCSR000BRT | ENCFF275XNU | CBX3 |
| ENCSR000EFS | ENCFF905NMP | JUN |
| ENCSR024CNP | ENCFF522KCK | EGR1 |
| ENCSR000BLO | ENCFF554UPA | GABPA |
| ENCSR670FDA | ENCFF843LQG | NFATC3 |
| ENCSR000EZW | ENCFF058LTT | JUN |
| ENCSR000EGB | ENCFF992BIU | TBL1XR1 |
| ENCSR621ATC | ENCFF046NMM | ZNF184 |
| ENCSR000EFW | ENCFF729POI | UBTF |
| ENCSR744WOO | ENCFF619AEN | TCF12 |
| ENCSR000BKR | ENCFF824ANA | POLR2AphosphoS5 |
| ENCSR798ILC | ENCFF032MJB | NCOR1 |
| ENCSR563LLO | ENCFF634VUQ | E2F1 |
| ENCSR695EQB | ENCFF535ZFM | ZNF24 |
| ENCSR325RLL | ENCFF779AZS | POLR2B |
| ENCSR041AXL | ENCFF951EDR | RFX1 |
| ENCSR115BBC | ENCFF151TIK | ASH1L |
| ENCSR000FBC | ENCFF443IZC | STAT2 |
| ENCSR635EXI | ENCFF529AJE | ZSCAN29 |
| ENCSR000EFY | ENCFF272CWK | ARID3A |
| ENCSR000FCB | ENCFF830UEH | MITF |
| ENCSR000ATW | ENCFF346SWX | CBX8 |
| ENCSR000BRS | ENCFF056DZM | NR2F2 |
| ENCSR563YDA | ENCFF428RTI | HDGF |
| ENCSR931HNY | ENCFF371FZO | NCOA1 |
| ENCSR000EGW | ENCFF662LUH | SMC3 |
| ENCSR155KHM | ENCFF805MAQ | ARNT |
| ENCSR502OEK | ENCFF667LOH | ELF1 |
| ENCSR000EGV | ENCFF819MHF | BHLHE40 |
| ENCSR076YPO | ENCFF666MKU | RNF2 |
| ENCSR948VFL | ENCFF589RYN | IKZF1 |
| ENCSR426MDV | ENCFF710ZFM | MIER1 |
| ENCSR820GND | ENCFF966CGZ | RNF2 |
| ENCSR352BJL | ENCFF772WLM | ZNF318 |
| ENCSR000EFP | ENCFF567XKS | ZNF384 |
| ENCSR031TFS | ENCFF909KNE | POLR2A |
| ENCSR000FAU | ENCFF304OMG | STAT1 |
| ENCSR000AQJ | ENCFF235UDJ | SAP30 |
| ENCSR000EGD | ENCFF416UYQ | BACH1 |
| ENCSR638QHV | ENCFF136UPB | ELF4 |
| ENCSR718SDE | ENCFF453DGG | RLF |
| ENCSR832OGB | ENCFF870ESB | LEF1 |
| ENCSR000EHP | ENCFF365NCW | POLR2A |
| ENCSR120MPG | ENCFF041AYU | PRDM10 |
| ENCSR914NEI | ENCFF678KKB | MTA3 |
| ENCSR000EGH | ENCFF318SAW | JUN |
| ENCSR085QEV | ENCFF023PLP | NBN |
| ENCSR249BHQ | ENCFF277ZUZ | ZNF592 |
| ENCSR797SWM | ENCFF105CED | MITF |
| ENCSR168CEE | ENCFF601ZZJ | NCOA6 |
| ENCSR760UVO | ENCFF512PBA | KLF16 |
| ENCSR547LKC | ENCFF334TMJ | GATAD2B |
| ENCSR000FCE | ENCFF452INW | ETV6 |
| ENCSR159OCC | ENCFF539RBU | 3xFLAG-ATF1 |
| ENCSR024LKA | ENCFF608YGZ | HDAC3 |
| ENCSR788RSW | ENCFF251SPU | SOX6 |
| ENCSR998AJK | ENCFF762NRN | NRF1 |
| ENCSR000BKS | ENCFF937JNR | TAF1 |
| ENCSR633EIC | ENCFF270VUX | 3xFLAG-PBX2 |
| ENCSR715CCR | ENCFF315QEI | DPF2 |
| ENCSR119VCX | ENCFF552BXT | PHF21A |
| ENCSR000BQY | ENCFF613YPH | PML |
| ENCSR000DWE | ENCFF389ELU | CTCF |
| ENCSR221GAN | ENCFF768YPD | MBD2 |
| ENCSR000EGX | ENCFF216COR | MAFK |
| ENCSR145TSJ | ENCFF771LVW | ATF4 |
| ENCSR697YLJ | ENCFF845OPG | CBFA2T3 |
| ENCSR160QYK | ENCFF407WIN | GATAD2A |
| ENCSR699PVC | ENCFF317SWK | CBFA2T2 |
| ENCSR000EGI | ENCFF003UXM | MAFF |
| ENCSR669NFS | ENCFF108FQP | ARNT |
| ENCSR000BMV | ENCFF503LMD | FOSL1 |
| ENCSR431XGJ | ENCFF091EWT | PYGO2 |
| ENCSR171CAY | ENCFF910WWV | E2F7 |
| ENCSR395HWC | ENCFF214VWR | IKZF1 |
| ENCSR898XMH | ENCFF804BSI | ZFP91 |
| ENCSR675LRO | ENCFF745CJF | MLLT1 |
| ENCSR030TJP | ENCFF326QUT | DACH1 |
| ENCSR385AHH | ENCFF941XDH | ZNF24 |
| ENCSR004GKA | ENCFF892BNB | ZEB2 |
| ENCSR654CQU | ENCFF117NUA | SNIP1 |
| ENCSR863KUB | ENCFF357AHH | TCF7 |
| ENCSR807BGP | ENCFF843MEU | MTA1 |
| ENCSR107GRP | ENCFF037UMP | MLLT1 |
| ENCSR113LAS | ENCFF299JOC | MTA2 |
| ENCSR869IUD | ENCFF207FUE | ATF2 |
| ENCSR000EGO | ENCFF436JKE | RFX5 |
| ENCSR389PWB | ENCFF007LEG | ZBTB5 |
| ENCSR000EHJ | ENCFF122XOC | STAT1 |
| ENCSR124BJR | ENCFF569AQR | ETV6 |
| ENCSR000EHN | ENCFF993YKH | SMARCB1 |
| ENCSR197ALX | ENCFF086TFH | HDGF |
| ENCSR000EGF | ENCFF027TJN | POLR2AphosphoS2 |
| ENCSR670JDQ | ENCFF124NBN | RB1 |
| ENCSR594SMP | ENCFF762GKR | PHF20 |
| ENCSR657EOF | ENCFF594KPG | NFRKB |
| ENCSR876GXA | ENCFF909FRB | ZBTB33 |
| ENCSR920BLG | ENCFF446MUL | SIN3A |
| ENCSR000EVY | ENCFF821JKR | TRIM28 |
| ENCSR888XZK | ENCFF749WKK | TCF7L2 |
| ENCSR102KIN | ENCFF429TTF | ZMYM3 |
| ENCSR000BLI | ENCFF160TXQ | E2F6 |
| ENCSR000BMD | ENCFF617WYS | ELF1 |
| ENCSR343IFJ | ENCFF259WDV | CC2D1A |
| ENCSR474CVP | ENCFF580SYA | TRIM28 |
| ENCSR000FAZ | ENCFF695KNI | MYC |
| ENCSR000EGQ | ENCFF897LDW | NFYB |
| ENCSR737LTZ | ENCFF423GUZ | MYNN |
| ENCSR822CCM | ENCFF771TNK | ARID1B |
| ENCSR100UQX | ENCFF449ZBD | TAF9B |
| ENCSR315NNL | ENCFF146FGH | CHAMP1 |
| ENCSR000FCD | ENCFF548ITQ | SMAD5 |
| ENCSR000EGA | ENCFF117UOA | TBL1XR1 |
| ENCSR000EWL | ENCFF281NFW | E2F4 |
| ENCSR707QWA | ENCFF951LYB | NR2F6 |
| ENCSR137ZMQ | ENCFF418BWE | REST |
| ENCSR000BME | ENCFF482PBU | ZBTB7A |
| ENCSR033NQK | ENCFF696WVL | ZNF830 |
| ENCSR731LHZ | ENCFF233MGP | E4F1 |
| ENCSR803EKW | ENCFF989AVX | NCOA2 |
| ENCSR387SYS | ENCFF389KBV | DEAF1 |
| ENCSR494TDU | ENCFF460GYT | NRF1 |
| ENCSR000BNW | ENCFF593FWV | SIX5 |
| ENCSR583ACG | ENCFF825ZCR | BRD4 |
| ENCSR742IDN | ENCFF477IDC | NR2C1 |
| ENCSR968GIB | ENCFF667ANE | RFX1 |
| ENCSR000EFZ | ENCFF161BNR | UBTF |
| ENCSR928KOR | ENCFF058FJN | GMEB1 |
| ENCSR000EZV | ENCFF120IMP | MYC |
| ENCSR302AWT | ENCFF154SRU | FOXK2 |
| ENCSR349TZO | ENCFF097AKG | NCOA2 |
| ENCSR000EHO | ENCFF714WNT | SMARCA4 |
| ENCSR742TMU | ENCFF337XJU | ZNF282 |
| ENCSR710WLO | ENCFF557SFL | MGA |
| ENCSR972ZBV | ENCFF953PPE | ATF7 |
| ENCSR000EWF | ENCFF632ZHY | YY1 |
| ENCSR331GDC | ENCFF059AZP | ZBTB11 |
| ENCSR508DQA | ENCFF606WYG | FOXK2 |
| ENCSR720HUL | ENCFF784XVN | E2F1 |
| ENCSR588AKU | ENCFF049YPY | RUNX1 |
| ENCSR429QPP | ENCFF805OCR | FOXM1 |
| ENCSR970NKQ | ENCFF067JKB | NR2F1 |
| ENCSR028UIU | ENCFF011BHF | ATF3 |
| ENCSR524BUE | ENCFF691HAG | RAD51 |
| ENCSR795IYP | ENCFF529CTW | JUNB |
| ENCSR796ITY | ENCFF132TXF | NFIC |
| ENCSR907MZR | ENCFF263NDI | TRIM24 |
| ENCSR000EVX | ENCFF390BXU | ZNF274 |
| ENCSR780BBJ | ENCFF129QWO | ZZZ3 |
| ENCSR000BPJ | ENCFF101MTI | CTCF |
| ENCSR121PFY | ENCFF575VHZ | CDC5L |
| ENCSR162IEM | ENCFF061UNO | MYBL2 |
| ENCSR065XVO | ENCFF077WVW | CHAMP1 |
| ENCSR991ELG | ENCFF440HZQ | SP1 |
| ENCSR000EWN | ENCFF607PKH | ZNF263 |
| ENCSR882ERE | ENCFF329HLY | ZKSCAN1 |
| ENCSR272JAT | ENCFF525FJA | CBX5 |
| ENCSR077DKV | ENCFF159OAX | CREM |
| ENCSR486IFJ | ENCFF038PPG | ESRRA |
| ENCSR948QLZ | ENCFF191MYC | CBX1 |
| ENCSR642VZY | ENCFF561ZCZ | KDM4B |
| ENCSR322CFO | ENCFF319TPR | ZEB2 |
| ENCSR038DJJ | ENCFF012KDB | SMAD1 |
| ENCSR106FRG | ENCFF412EVD | TAL1 |
| ENCSR757IIU | ENCFF912CZG | HMBOX1 |
| ENCSR000BLP | ENCFF448ZSO | MAX |
| ENCSR000EGP | ENCFF086USH | ZNF143 |
| ENCSR115SMW | ENCFF804ARW | PKNOX1 |
| ENCSR211LTF | ENCFF237YNN | EGR1 |
| ENCSR000FAX | ENCFF716CWM | POLR2A |
| ENCSR138YYY | ENCFF589ZQU | GABPB1 |
| ENCSR000EFN | ENCFF734CJH | HCFC1 |
| ENCSR000EZU | ENCFF723QGS | MYC |
| ENCSR000EHE | ENCFF196UZE | CEBPB |
| ENCSR000EHA | ENCFF609UTS | TBP |
| ENCSR075HTM | ENCFF672AGD | HDAC2 |
| ENCSR000EGL | ENCFF736MBF | IRF1 |
| ENCSR910JAI | ENCFF577EBU | NCOR1 |
| ENCSR439OCL | ENCFF776NRP | ZNF407 |
| ENCSR167KBO | ENCFF195JOB | ZNF316 |
| ENCSR200JYP | ENCFF647FMI | ZNF316 |
| ENCSR000BKT | ENCFF409EYL | USF1 |
| ENCSR497VFH | ENCFF947YGS | ZNF639 |
| ENCSR546IHU | ENCFF352VEC | ZNF184 |
| ENCSR414TYY | ENCFF264QLP | RUNX1 |
| ENCSR000BRQ | ENCFF712GEQ | CEBPB |
| ENCSR177XCS | ENCFF313PGK | BRD9 |
| ENCSR000BKU | ENCFF371NVU | YY1 |
| ENCSR957LDM | ENCFF857AXB | TRIM24 |
| ENCSR230PTV | ENCFF830BRF | ZBTB2 |
| ENCSR519WMW | ENCFF102TZK | SMARCC2 |
| ENCSR608XTF | ENCFF389LKZ | RNF2 |
| ENCSR157TCS | ENCFF355IGX | SMARCE1 |
| ENCSR370NFS | ENCFF705FAV | ZNF280A |
| ENCSR996ESX | ENCFF847JCH | NFRKB |
| ENCSR000BMR | ENCFF757ZGV | POLR2A |
| ENCSR658WFQ | ENCFF852MFA | NCOA1 |
| ENCSR000EGT | ENCFF548PPV | IRF1 |
| ENCSR000EWM | ENCFF484BSF | GATA1 |
| ENCSR000BRR | ENCFF916OUG | STAT5A |
| ENCSR175SZH | ENCFF148GRM | ZSCAN29 |
| ENCSR000EFT | ENCFF875JHB | GATA1 |
| ENCSR924GXX | ENCFF111EXG | PHB2 |
| ENCSR587OQL | ENCFF333IXA | SMARCA4 |
| ENCSR000EHB | ENCFF563DQA | TAL1 |
| ENCSR000BLR | ENCFF643PFS | SIN3A |
| ENCSR851BNE | ENCFF023KAG | MEIS2 |
| ENCSR138FUZ | ENCFF876CPX | RNF2 |
| ENCSR286PCG | ENCFF619KWZ | ZBED1 |
