# Supplementary material for: Decoding regulatory associations of G-quadruplex with epigenetic and transcriptomic functional components
Source: Front Genet. 2022 Aug 25;13:957023. doi: 10.3389/fgene.2022.957023 (PMC9452811; doi:10.3389/fgene.2022.957023)
Supplement: Supplementary file 3 [file Table2.docx]

**Supplementary Table 2 Jaccard scores for 205 TFs with three groups of G4s, separately.**

| TF | G4-I | G4-II | G4-III |
| --- | --- | --- | --- |
| SP1 | 0.046897 | 0.1512 | 0.002769 |
| IRF1 | 0.059324 | 0.145552 | 0.003285 |
| E2F6 | 0.063993 | 0.143817 | 0.006285 |
| ZBTB7A | 0.050922 | 0.143291 | 0.012605 |
| TAF1 | 0.061609 | 0.141963 | 0.005188 |
| NRF1 | 0.078723 | 0.132762 | 0.009321 |
| MAX | 0.087429 | 0.131031 | 0.009624 |
| TBP | 0.060008 | 0.126947 | 0.00361 |
| MYC | 0.083861 | 0.126095 | 0.00777 |
| UBTF | 0.062252 | 0.124893 | 0.007179 |
| POLR2A | 0.073765 | 0.124575 | 0.01263 |
| RB1 | 0.06296 | 0.122815 | 0.004056 |
| E2F4 | 0.042206 | 0.112835 | 0.001376 |
| SMAD5 | 0.049302 | 0.111424 | 0.004308 |
| RBBP5 | 0.052357 | 0.109787 | 0.007401 |
| CREM | 0.077869 | 0.108658 | 0.005122 |
| MTA3 | 0.069196 | 0.10716 | 0.004481 |
| SIN3A | 0.042933 | 0.107062 | 0.005012 |
| CTBP1 | 0.075133 | 0.106762 | 0.006055 |
| GMEB1 | 0.064786 | 0.106639 | 0.007192 |
| ELF1 | 0.058211 | 0.105823 | 0.004752 |
| EGR1 | 0.05294 | 0.104677 | 0.012984 |
| POLR2AphosphoS5 | 0.059157 | 0.10435 | 0.004657 |
| MNT | 0.067524 | 0.104165 | 0.007193 |
| GABPB1 | 0.062845 | 0.102072 | 0.00798 |
| ATF7 | 0.073225 | 0.101669 | 0.005475 |
| SKIL | 0.061231 | 0.101644 | 0.003729 |
| HCFC1 | 0.054914 | 0.100747 | 0.001717 |
| CREB3L1 | 0.055884 | 0.098232 | 0.002954 |
| L3MBTL2 | 0.068686 | 0.094804 | 0.007609 |
| MBD2 | 0.053727 | 0.094725 | 0.002233 |
| HDAC1 | 0.069331 | 0.088749 | 0.009866 |
| POLR2B | 0.044237 | 0.088133 | 0.004338 |
| GABPA | 0.047893 | 0.087521 | 0.002193 |
| BHLHE40 | 0.061467 | 0.085891 | 0.003653 |
| ZNF639 | 0.04667 | 0.085267 | 0.003683 |
| PML | 0.054166 | 0.081871 | 0.003494 |
| NEUROD1 | 0.046404 | 0.080691 | 0.001628 |
| ESRRA | 0.055267 | 0.080188 | 0.003721 |
| CBX1 | 0.035873 | 0.078298 | 0.002636 |
| E2F1 | 0.051546 | 0.076494 | 0.003754 |
| PHF20 | 0.040685 | 0.075962 | 0.00356 |
| JUND | 0.067305 | 0.07533 | 0.003917 |
| HDAC2 | 0.060487 | 0.07403 | 0.006773 |
| ZNF282 | 0.031971 | 0.073153 | 0.002459 |
| KDM4B | 0.027514 | 0.073142 | 0.002022 |
| REST | 0.062853 | 0.072898 | 0.004919 |
| E4F1 | 0.060203 | 0.07213 | 0.004107 |
| SIN3B | 0.030095 | 0.071971 | 0.00194 |
| YY1 | 0.034696 | 0.071828 | 0.001986 |
| FOXK2 | 0.056313 | 0.069245 | 0.004417 |
| MXI1 | 0.036713 | 0.069147 | 0.0015 |
| NCOA1 | 0.056767 | 0.067273 | 0.004351 |
| SOX6 | 0.055974 | 0.067091 | 0.003266 |
| TBL1XR1 | 0.055016 | 0.066947 | 0.002229 |
| SAP30 | 0.030292 | 0.066933 | 0.003634 |
| E2F8 | 0.041005 | 0.066087 | 0.001969 |
| ZBTB2 | 0.041581 | 0.065545 | 0.003093 |
| TAF9B | 0.046873 | 0.065374 | 0.003449 |
| PRDM10 | 0.041861 | 0.063725 | 0.006207 |
| ELF4 | 0.049028 | 0.063521 | 0.003309 |
| NFRKB | 0.042899 | 0.06259 | 0.005609 |
| NCOR1 | 0.052115 | 0.06243 | 0.005522 |
| RNF2 | 0.045551 | 0.061948 | 0.002539 |
| ZBTB40 | 0.040651 | 0.060473 | 0.005121 |
| MGA | 0.047422 | 0.059208 | 0.006602 |
| JUN | 0.050117 | 0.057533 | 0.002445 |
| PKNOX1 | 0.04839 | 0.057454 | 0.005446 |
| ZNF24 | 0.048583 | 0.056746 | 0.005924 |
| NBN | 0.044917 | 0.05671 | 0.002871 |
| CBX3 | 0.047966 | 0.055868 | 0.00255 |
| GATAD2B | 0.022874 | 0.054882 | 0.00202 |
| GATAD2A | 0.032157 | 0.054732 | 0.003194 |
| SMAD1 | 0.031081 | 0.054422 | 0.001256 |
| MEIS2 | 0.046753 | 0.053752 | 0.003365 |
| MYNN | 0.043992 | 0.053617 | 0.002151 |
| ZNF592 | 0.045687 | 0.05315 | 0.004525 |
| EP300 | 0.048875 | 0.05299 | 0.002489 |
| ZNF143 | 0.049535 | 0.051739 | 0.002732 |
| THAP1 | 0.020935 | 0.051429 | 0.00088 |
| NFIC | 0.045134 | 0.051387 | 0.006364 |
| MLLT1 | 0.038154 | 0.051333 | 0.004861 |
| ZKSCAN1 | 0.037029 | 0.051207 | 0.001707 |
| HDGF | 0.031221 | 0.050565 | 0.003838 |
| ZEB2 | 0.050218 | 0.049321 | 0.004471 |
| RLF | 0.027055 | 0.048774 | 0.002276 |
| BRD4 | 0.022229 | 0.047385 | 0.002987 |
| TEAD4 | 0.04898 | 0.046783 | 0.003253 |
| NR2F1 | 0.043157 | 0.0455 | 0.005379 |
| LEF1 | 0.038675 | 0.045281 | 0.003023 |
| NR2F2 | 0.042876 | 0.045279 | 0.002541 |
| CTCF | 0.045353 | 0.045208 | 0.005559 |
| AFF1 | 0.039986 | 0.044698 | 0.003441 |
| NFYB | 0.020853 | 0.044297 | 0.000918 |
| TRIM24 | 0.041368 | 0.043809 | 0.004149 |
| ZBED1 | 0.025059 | 0.043774 | 0.001157 |
| DPF2 | 0.046487 | 0.04345 | 0.006049 |
| MTA1 | 0.035138 | 0.042921 | 0.002695 |
| USF1 | 0.025533 | 0.042008 | 0.001749 |
| SMARCA4 | 0.044664 | 0.041199 | 0.004725 |
| RUNX1 | 0.024311 | 0.039878 | 0.000762 |
| SMARCE1 | 0.040188 | 0.039834 | 0.004694 |
| NR2C1 | 0.033668 | 0.039709 | 0.002976 |
| IKZF1 | 0.040441 | 0.038909 | 0.010141 |
| TCF7 | 0.024451 | 0.038665 | 0.000923 |
| ARID1B | 0.04321 | 0.0386 | 0.005992 |
| ZBTB11 | 0.024634 | 0.038297 | 0.001584 |
| KLF16 | 0.040984 | 0.038194 | 0.002234 |
| ELK1 | 0.021433 | 0.037475 | 0.000612 |
| SMC3 | 0.038975 | 0.037306 | 0.002669 |
| MITF | 0.028868 | 0.037069 | 0.003018 |
| BRD9 | 0.029366 | 0.036853 | 0.002734 |
| DEAF1 | 0.017009 | 0.036812 | 0.000717 |
| ZFP91 | 0.021785 | 0.03648 | 0.003038 |
| CTCFL | 0.022921 | 0.036117 | 0.001257 |
| ARNT | 0.039811 | 0.035158 | 0.002858 |
| IRF2 | 0.023349 | 0.035111 | 0.000921 |
| CC2D1A | 0.041912 | 0.035085 | 0.003467 |
| FOXM1 | 0.032779 | 0.034914 | 0.001893 |
| ZSCAN29 | 0.026402 | 0.034372 | 0.003125 |
| POLR2AphosphoS2 | 0.018388 | 0.033667 | 0.001971 |
| BCOR | 0.029918 | 0.033349 | 0.005195 |
| RAD51 | 0.023347 | 0.03334 | 0.001705 |
| DACH1 | 0.027099 | 0.032411 | 0.002043 |
| TAL1 | 0.037483 | 0.031873 | 0.003437 |
| NFYA | 0.014505 | 0.030828 | 0.000316 |
| CBFA2T3 | 0.037329 | 0.030442 | 0.005155 |
| NFATC3 | 0.030005 | 0.029843 | 0.001822 |
| 3xFLAG-ATF1 | 0.013145 | 0.029711 | 0.000321 |
| ASH1L | 0.016327 | 0.029582 | 0.000789 |
| CBFA2T2 | 0.035129 | 0.029083 | 0.00374 |
| C11orf30 | 0.03065 | 0.02879 | 0.003199 |
| HES1 | 0.022254 | 0.02855 | 0.00129 |
| PHF21A | 0.016083 | 0.028423 | 0.001194 |
| SREBF1 | 0.013186 | 0.027424 | 0.000313 |
| ZNF384 | 0.024139 | 0.027249 | 0.002048 |
| ZNF184 | 0.026496 | 0.027181 | 0.002567 |
| RFX1 | 0.020416 | 0.026792 | 0.004386 |
| ZNF263 | 0.012708 | 0.025185 | 0.002852 |
| TCF12 | 0.033755 | 0.025084 | 0.003308 |
| NFE2 | 0.029881 | 0.025018 | 0.001965 |
| ZHX1 | 0.014849 | 0.024845 | 0.000376 |
| TRIM28 | 0.028168 | 0.024342 | 0.001614 |
| NR2F6 | 0.025572 | 0.023443 | 0.002792 |
| MTA2 | 0.027618 | 0.02325 | 0.002203 |
| SETDB1 | 0.014262 | 0.023085 | 0.00071 |
| SMARCC2 | 0.02884 | 0.02293 | 0.001865 |
| ZNF407 | 0.013647 | 0.022844 | 0.00144 |
| NCOA6 | 0.010158 | 0.022398 | 0.00124 |
| RCOR1 | 0.02082 | 0.021984 | 0.000783 |
| ZNF318 | 0.022493 | 0.02144 | 0.001465 |
| ETV6 | 0.018261 | 0.021432 | 0.001468 |
| MIER1 | 0.013645 | 0.020472 | 0.002255 |
| CBX5 | 0.015155 | 0.020449 | 0.000549 |
| CEBPB | 0.022607 | 0.020044 | 0.003134 |
| ATF3 | 0.025304 | 0.019459 | 0.003792 |
| GATA1 | 0.026285 | 0.01934 | 0.000971 |
| ARID3A | 0.022185 | 0.018253 | 0.00107 |
| E2F7 | 0.010464 | 0.017738 | 0.000401 |
| 3xFLAG-PBX2 | 0.009507 | 0.017452 | 0.000487 |
| STAT1 | 0.01386 | 0.017212 | 0.000502 |
| RFX5 | 0.009471 | 0.017152 | 0.000327 |
| ATF2 | 0.017636 | 0.01675 | 0.002212 |
| USF2 | 0.009532 | 0.016686 | 0.000361 |
| CHAMP1 | 0.021377 | 0.016056 | 0.001346 |
| STAT5A | 0.024355 | 0.015863 | 0.000888 |
| JUNB | 0.012848 | 0.015842 | 0.000584 |
| ZBTB5 | 0.010211 | 0.015557 | 0.001326 |
| SMARCA5 | 0.012409 | 0.015413 | 0.002609 |
| MAFK | 0.021249 | 0.014752 | 0.001285 |
| ARID2 | 0.018335 | 0.014622 | 0.001497 |
| FOSL1 | 0.015933 | 0.014008 | 0.000912 |
| ATF4 | 0.017511 | 0.013889 | 0.003133 |
| TCF7L2 | 0.007441 | 0.013861 | 0.000248 |
| HDAC3 | 0.01875 | 0.013785 | 0.000938 |
| ZBTB33 | 0.018335 | 0.013674 | 0.004752 |
| HMBOX1 | 0.023693 | 0.013257 | 0.002487 |
| EHMT2 | 0.021676 | 0.012868 | 0.002282 |
| BACH1 | 0.009514 | 0.012323 | 0.000383 |
| GATA2 | 0.018285 | 0.012248 | 0.000525 |
| CDC5L | 0.007502 | 0.011925 | 0.001181 |
| BMI1 | 0.005749 | 0.011456 | 0.000326 |
| SIX5 | 0.008593 | 0.011442 | 0.00014 |
| NUFIP1 | 0.008541 | 0.011271 | 0.000357 |
| ZZZ3 | 0.008475 | 0.011034 | 0.00038 |
| CUX1 | 0.013065 | 0.010748 | 0.000643 |
| ZNF316 | 0.015632 | 0.010369 | 0.00348 |
| MYBL2 | 0.007738 | 0.009066 | 0.000434 |
| PHB2 | 0.012661 | 0.008496 | 0.00102 |
| SMARCB1 | 0.006035 | 0.008338 | 0.000407 |
| NFXL1 | 0.00439 | 0.008049 | 0.002614 |
| ZNF830 | 0.005608 | 0.007804 | 0.000584 |
| SNIP1 | 0.004308 | 0.007776 | 0.000371 |
| ZMYM3 | 0.010195 | 0.007051 | 0.003364 |
| STAT2 | 0.005829 | 0.006613 | 0.000259 |
| NCOA2 | 0.01081 | 0.006397 | 0.000493 |
| ARHGAP35 | 0.004065 | 0.006375 | 0.000781 |
| MAFF | 0.012335 | 0.006278 | 0.001079 |
| PYGO2 | 0.004502 | 0.005516 | 0.000488 |
| ZNF274 | 0.004553 | 0.005057 | 0.000206 |
| THRAP3 | 0.002827 | 0.001805 | 0.000352 |
| TAF7 | 0.000935 | 0.001726 | 5.7E-05 |
| MEF2A | 0.001748 | 0.00172 | 0.000119 |
| ZNF280A | 0.001334 | 0.000843 | 0.000531 |
| CBX8 | 0.000121 | 7.55E-05 | 0.000404 |
